# Antitumor and antimetastasis effects of carboplatin liposomes with polyethylene glycol-2000 on SGC-7901 gastric cell-bearing nude mice

**DOI:** 10.3892/ol.2014.2494

**Published:** 2014-09-03

**Authors:** JIANZHONG ZHANG, CHANGMING HUANG, HEGUANG HUANG

**Affiliations:** 1 Department of Gastrosurgery, Fujian Medical University Union Hospital, Fuzhou, Fujian 350001, P.R. China; 2 The Basic Surgical Department, Fujian Medical University Union Hospital, Fuzhou, Fujian 350001, P.R. China

**Keywords:** antitumor effect, antimetastatic effect, carboplatin liposome, nude mice

## Abstract

The present study aimed to analyze the characteristics of polyethylene glycol (PEG)ylated carboplatin liposomes (PL-CBPs), including size, stability, their release, entrapping and loading efficiencies, and their antitumor and antimetastatic effects on the lymph nodes. The PL-CBPs were prepared using PEG-2000 with the thin film hydration method. The liposome size and release, entrapping and loading efficiencies were detected by ultra-violet/visible spectrophotometry. A nude mouse model was established with the SGC-7901 gastric cell line to evaluate the antitumor effect of the PL-CBP. After 7 days, the mice were randomly divided into three groups (the control, CBP, and PL-CBP groups). CBP and PL-CBP were administered at a dose of 10 mg/kg for two consecutive cycles of treatment, 5 days apart, to their respective groups. In each group, two doses of 5 mg/kg were administered every 48 h. The tumor weight and volume were detected, and the food intake and body weight were measured during the administration. Apoptosis in the tumor cells was evaluated by terminal deoxynucleotidyl transferase-mediated dUTP nick end labeling and platinum (Pt) accumulation was detected by atomic absorption spectroscopy. Lastly, lymph node metastasis was evaluated by hematoxylin and eosin staining. The PL-CBPs were more stable when comapred with CBP alone, and the drug release efficiency was 0.7, 22.5, 48.7 and 65.1% at 37°C for 0, 12, 24 and 48 h. The results showed that the encapsulation efficiency was 85% and the loading efficiency was 0.15 mg/mg lipid. After 35 days, PL-CBP induced potent antitumor effects compared with the control and CBP groups (PL-CBP vs. control, P<0.01; PL-CBP vs. CBP, P<0.05). PL-CBP and CBP induced a lower and the lowest body weight and level of food intake, respectively, compared with the control group (CBP vs. control, P<0.05). The apoptosis rate and lymph node metastasis in the PL-CBP group was higher than that in the CBP and control groups (PL-CBP vs. control, P<0.01; PL-CBP vs. CBP, P<0.05). Pt accumulation in the tumors was higher in the PL-CBP group than in the CBP group (PL-CBP vs. CBP, P<0.05). The PL-CBPs were more stable in the circulation and could be released more slowly at the tumor site than compared with CBP injection. The PL-CBPs showed potent antitumor and antimetastatic effects on the lymph nodes.

## Introduction

Gastric cancer is the second most common cause of cancer-related mortality worldwide and is difficult to cure in Western countries, mainly since the majority of patients present with advanced disease (1). According to the World Health Organization, >100 million individuals are diagnosed worldwide every year and the three-year retrospective survey of gastric cancer shows the rate to be 23.2% in China (2). Malignancy is one of the most significant biological characteristics of gastric cancer. In total, >90% of gastric cancer patients suffer metastasis in different organs, including the lymph nodes, liver and lungs (3). Lymph node metastasis is closely associated with the five-year survival rate of gastric cancer. Certain studies have shown that the five-year survival rate drops to 30.5% in patients with lymph node metastasis and rises to 86.1% in those without lymph node metastasis (4–5).

Surgery is the most commonly used treatment for gastric tumors, but >50% of patients cannot rely on surgery to remove all lesions, as their lymph node metastasis has progressed outside the scope of surgical resection (6). At present, chemotherapy is the conventional treatment for lymph node metastases, but it is of detriment that the effect of chemotherapy drugs is not obvious and that the high plasma concentrations often produce serious side-effects. Therefore, the search for effective chemotherapy drugs for gastric cancer to prevent lymphatic metastasis, improve therapeutic efficacy and reduce toxicity is extremely important (7).

Certain studies have shown that liposomes as drug carriers can have affinity for tumor cells, changing the distribution of drugs in tissues and selectively killing tumor cells or restraining their reproduction (8). Liposomes are considered to be efficient carriers due to certain advantages, including their ability to incorporate water and lipid soluble agents, the presence of a fluid liposomal membrane giving high versatility, their size and their superficial charge (8). Therefore, liposomes are able to improve therapeutic efficacy and reduce the side-effects. Sterically stabilized liposomes are generated as a result of the insertion of polyethylene glycol (PEG)-derived phospholipids into the liposomal membrane (9–12).

Carboplatin CBP; cis-diammine (1,1-cyclobutanedicarboxylato)-platinum (II)] is recommended for the chemotherapy of numerous types of cancer, including gastric tumors. Standard second-line regimens often include CBP, as it exhibits non-cross resistance. However, the side-effects of CBP include dose-related myelosuppression, with severe thrombocytopenia and leucopenia. Thus, the ability to prepare PL-CBPs is important to reduce or prevent its side-effects and improve its target effect. Certain studies have reported that PL-CBPs coated with L-α egg phosphatidylcholine and *O*-palmitoylpullulan enhance the therapeutic effect and the stability of storage conditions. However, there are no studies on the effect of PL-CBPs on lymph node metastasis in a gastric cancer mouse model (13,14).

In the present study, PL-CBPs were prepared with PEG-2000 and the PL-CBP characteristics, including their size and stability, their release, entrapping and loading efficiencies, and their antitumor and antimetastatic effects on the lymph nodes, were analyzed.

## Materials and methods

CBP (purity, >99%) was purchased from Kunming Precious Metal Institute (Kunming, Yunnan, China). Injectable CBP (100 mg/bottle) was purchased from Jinan Qilu Pharmaceutical Company (Jinan, Shandong, China), while phosphatidylethanolamine (PE) and cholesterol were purchased from Shanghai Dongshang Company (Shanghai, China). PEG-2000 was purchased from Sigma-Aldrich (St. Louis, MO, USA). All other chemicals, unless otherwise stated, were obtained from Sigma-Aldrich.

### PL-CBP preparation

Liposomes containing CBP were prepared according to the thin film hydration method, as previously described (15). Briefly, lipids, including PE and cholesterol (PE:cholesterol, 4:1), were dissolved in 30 ml chloroform to form a mixture, and then the organic solvent was removed by using rotary evaporation under reduced pressure to obtain a film. The dry lipid film was hydrated with a solution of CBP dissolved in 5% glucose (2 mg/ml). The dispersion of the lipid was facilitated by mechanical agitation in an ultrasound bath for 1 min. The PEG-2000 dissolved in phosphate-buffered saline (PBS) with the prepared lipid in an equal volume were mixed and placed at 4°C for 60 min. Next, the same volume of PBS was added to the system at 4°C for 60 min.

### Drug release

This study was carried out at 37°C, as it was used for *in vitro* and *in vivo* studies. Next, 2 ml of formulation mixture solution in a dialysis bag was incubated in 5% glucose at 37°C, whilst being continuously agitated. Samples collected at different times (0, 12, 24 and 48 h) were ultrafiltered using the Genesys 10 ultra-violet/visible spectrophotometry (Thermo Genesys 10; Thermo Scientific, Waltham, MA, USA) at 235 nm. Release efficiency was expressed by the equation: Release efficiency (%) = (release CBP in liquid/total CBP in liposome) × 100. In addition, the particle size was also characterized in these samples.

### Determination of encapsulation efficiency

The detection of CBP was performed using an ultraviolet (UV) wavelength at 279 nm. In this assay, full wavelength scanning was used to detect the encapsulation efficiency. The first step was to detect and calculate the standard curve with the various concentrations of PL-CBP (0.01–0.25 mg/ml). The liposome samples were then diluted with sodium chloride injection fluid (0.9%). Next, the fluid was separated by gel exclusion chromatography over Sephadex G-50 (General Electric Company, Fairfield, CT, USA). The concentration of entrapped CBP was determined by Genesys 10 ultra-violet/visible spectrophotometry following lysis of the liposomes with anhydrous alcohol. The UV absorbance of the sample solution was measured at 235 nm. Each determination was made in triplicate. The encapsulation efficiency was expressed by the following equation: Encapsulation (%) = (QEn/Qtotal) × 100, where QEn is quantity of CBP (mg)/lipid (mg) determined following removal of free drug, and Qtotal is the quantity of CBP (mg)/lipid (mg) determined at the stage when the drug was added to the lipid film.

### Cell line

The human gastric cancer SGC-7901 cell line was obtained from the American Type Culture Collection (Manassas, VA, USA). The cells were grown in Dulbecco’s modified Eagle’s medium containing 10% fetal bovine serum, 100 U/ml penicillin and 100 μg/ml streptomycin in a 37°C humidified incubator with 5% CO_2_. The cells were subcultured at 80 to 90% confluence. The cells used in this study were subjected to ≤20 cell passages.

### Tumor xenograft

A total of 30 male BALB/c nude mice with body weights ranging between 18 and 22 g were injected with SGC-7901 cell line suspension in the right flank. After 7 days, the mice were randomly divided into three groups (the control, CBP and PL-CBP groups). CBP and PL-CBP were administered at 10 mg/kg for two consecutive cycles of treatment, 5 days apart, to their respective groups. In each group, two doses of 5 mg/kg were administered every 48 h. The dose of liposomes was calculated based on the quantity of encapsulated CBP (μg)/lipid (mg). At the end of the study, the tumors were excised and weighed. Tumor size was measured and volume was calculated according to the following formula: Tumor volume (mm^3^) = d^2^ × D/2, where d and D were the shortest and longest diameter, respectively. This study was approved by the ethics committee of Fujian Medical University (Fuzhou, China).

### Food intake and body weight

Each cage of mice was provided daily with food, and on the following day, the remainder was collected and weighed in order to calculate daily food intake. Body weight was measured at baseline and at the end of treatment.

### Hematoxylin and eosin (HE) staining

Histological examination was performed by HE staining. Tumor tissues were fixed with 10% buffered formalin for 24 h. Samples were then paraffin-embedded, sectioned and stained with HE. Histopathological changes were observed under a light microscope. According to the grading standards of lymph node metastasis, the metastasis was divided into the following four grades: 0, no tumor cells in the lymph nodes; +, numerous tumor cells at the edge of the sinus; ++, tumor cells invade the middle of the sinus; and +++, tumors occupy more than half of the cross-section in the lymph node (16).

### Analysis of Pt in tissue by atomic absorption spectroscopy (AAS)

The level of Pt was determined in the tissues by electrothermal atomization in an AAS 8000 graphite furnace (Renrui Company, Shanghai, China). The tissue samples were analyzed following enzymatic digestion and modification of the final matrix. The tissue was dried at 100°C for 20 h and weighed. Subsequent to wet combustion with 1.0 ml nitric acid and 0.5 ml perchloric acid, all samples were diluted to 10 ml with extra-pure water (Milli-Q, Nihon Millipore Kogyo KK, Tokyo, Japan), and the Pt content in the tissue was measured. The furnace conditions and instrumental parameters for the determination of Pt were 70–105°C for 60 sec, 1,400°C for 30 sec, 2,800°C for 10 sec and 2,900°C for 7 sec, and argon was used as a carrier gas at a rate of 200 ml/min. The results were expressed as the quantity of Pt (mg)/dried tissue (g).

### Tumor apoptosis

Tumor sections from the mice treated as aforementioned were fixed with 4% paraformaldehyde for 48 h. The 5-μm thick sections of the tumor samples were analyzed by terminal deoxynucleotidyl transferase-mediated dUTP nick end labeling (TUNEL) staining using a TumorTACS *in situ* Apoptosis kit (Roche Bioscience, Palo Alto, CA, USA) to detect fragmented DNA according to the manufacturer’s instructions. Microscopic immunohistochemical images were captured by an Olympus microscope (Olympus Corporation, Tokyo, Japan) and a moticam 5000C camera from Motic Instruments (Richmond, BC, Canada), and analyzed by Motic Med 6.0 software. The number of positive and total cells were counted at five arbitrarily selected microscopic fields at ×100 magnification (each 7,050 μm^2^ in size). A dark brown nucleus represented the positive apoptotic cells of the tumor, observed due to TUNEL staining, and the apoptosis index (AI) was calculated according to the following formula: AI = number of positive cells/total number of cells.

### Statistical analyses

Statistical analysis was performed with SPSS 16.0 (SPSS, Inc., Chicago, IL, USA). Data are presented as the mean ± standard deviation. A two-sided α level (type I error rate) of <0.05 was considered to indicate a statistically significant result. One way analysis of variance was used to analyze the data and Fisher’s protected least significant difference was used as a post hoc test.

## Results

### Stability analysis

The stability analysis results are shown in Table I. The size and distribution of the liposomes were relative to their stability. It was revealed that at 37°C, the lipsome size was similar at 0–48 h, as was expected. Once the liposomes had been incubated at 37°C for 0, 12, 24 and 48 h, the drug release efficiency was 0.7, 22.5, 48.7 and 65.1%. This effect represents an advantage of CBP, as the drug would be more stable in the blood circulation and could be released slowly at the tumor site.

### Antitumor effect

**Figure 1:**
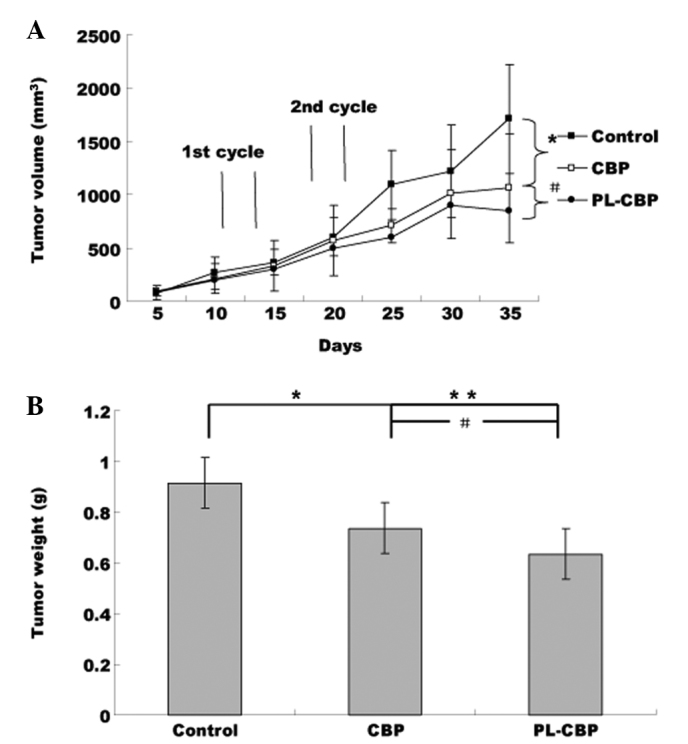
Antitumor effect of PL-CBP and CBP in SGC-7901 gastric tumor cell-bearing mice after 35 days. The antitumor effect of each regime was evaluated by measuring (A) tumor volume and (B) weight. Values are presented as the mean ± standard deviation (n=8). ^*^P<0.05 and ^**^P<0.01 vs. control; ^#^P<0.05 vs. CBP group. CBP, carboplatin; PL-CBP, polyethylene glycol (PEG)ylated CBP.

Fig. 1 shows that the PLs suppressed tumor growth more efficiently than the control. This inhibition reached statistical significance subsequent to the second cycle. At 35 days, compared with the control group, the tumor weight and tumor volume were reduced by 65% in the PL-CBP group (Fig. 1, P<0.01) and by 50% in the CBP group (P<0.05 vs. control; P<0.05 vs. PL-CBP).

### Average diet consumption and body weight

**Figure 2:**
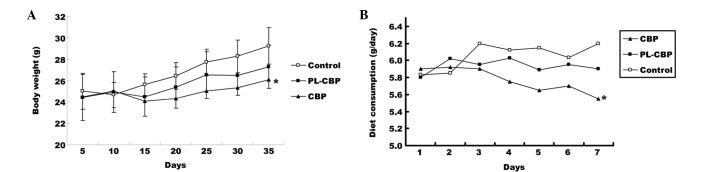
Effects of PL-CBP and CBP on body weight and food intake of SGC-7901 gastric tumor cell-bearing mice over 35 days. (A) Body weight and (B) average daily food intake were calculated. Values are presented as the mean ± standard deviation (n=8). ^*^P<0.05 vs. control. CBP, carboplatin; PL-CBP, polyethylene glycol (PEG)ylated CBP.

There was no significant difference in the body weight between the groups at baseline. The body weight per mouse ranged between 24 and 29 g after 35 days. Mice in the PL-CBP and CBP groups had a lower body weight compared with the control group, and this difference was significant in the CBP group compared with the control group (P<0.05; Fig. 2A). The average diet consumption was higher in the control group than in the PL-CBP and CBP groups and this difference was significant (P<0.05) between the CBP and control groups subsequent to the first cycle (Fig. 2B). The PL-CBP group appeared to gain fewer side-effects following treatment, while the CBP mice demonstrated impaired movement, ruffled fur and a greater loss of body weight.

### Tumor apoptosis

**Figure 3:**
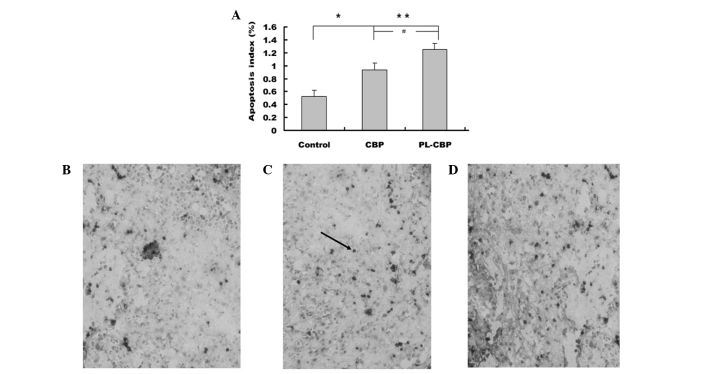
(A) Effects of PL-CBP and CBP on the apoptosis of the tumor cells. The apoptosis index is shown as a percentage of TUNEL-positive cells. Values are presented as the mean ± standard deviation (n=8). ^*^P<0.05 and ^**^P<0.01 vs. control; ^#^P<0.05 vs. CBP group. Immunohistochemical TUNEL staining of the (B) control, (C) CBP and (D) PL-CBP groups. Positive cells are indicated by arrows. CBP, carboplatin; PL-CBP, polyethylene glycol (PEG)ylated CBP; TUNEL, transferase-mediated dUTP nick end labeling.

In the present study, the results showed that the AI was significantly higher in the PL-CBP and CBP groups compared with the control group (P<0.05 and P<0.01, respectively). Representative images are shown in Fig. 3.

### Pt accumulation in the tumor

**Figure 4:**
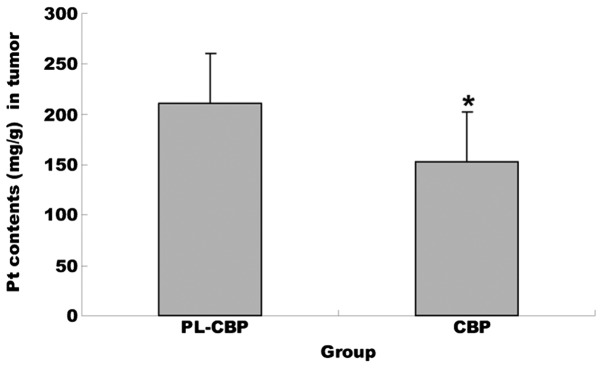
Pt content in the tumors following administration of PL-CBP and CBP. The Pt accumulation in the tumors was detected by atomic absorption spectroscopy, and values are presented as the mean ± standard deviation (n=8). ^*^P<0.05 vs. control. CBP, carboplatin; PL-CBP, polyethylene glycol (PEG)ylated CBP; Pt, platinum.

Pt concentrations in each sample were calculated by fitting linear regression lines to the points defined by the spiked concentration values and the corresponding integrated peak areas. In the tumor tissues, the concentration of Pt in the PL-CBP group was much greater than that accumulated from the injected form (P<0.01), which meant that the distribution of PL-CBP in the tissues was improved significantly (Fig. 4).

### Lymph node metastasis

The right submaxillary lymph node became swollen. The volume of lymph in the PL-CBP group was smaller than that in the CBP and control groups (PL-CBP vs. CBP, P<0.05; PL-CBP vs. control, P<0.01). The tumor migrated to the lymph in the control group, with grade +++ metastasis. The grade of metastasis in the PL-CBP and CBP groups was 0/+ and +/++ (Table II; PL-CBP vs. CBP, P<0.05; PL-CBP vs. control, P<0.01).

## Discussion

In the present study, a novel formula of PL-CBP was developed in liposomal dosage form to improve its delivery characteristics and inhibit the side-effects. Once CBP had been encapsulated in the liposomes with PEG-2000, it exhibited a high stability. Associated studies have indicated that PEG-modified chemotherapy drugs can overcome any limitations, including short circulation time and side-effects (16). The PEG-modification can confer significant benefits on drugs, such as increasing their solubility by the association with water molecules. The increased molecular weight of the PEG-modified complex reduces the speed of kidney clearance and induces few side-effects (17). Other than the use of the PEG-2000 as a modifier of chemotherapy drugs, various lipids have been widely used to form liposomes, including *O*-palmitoylpullulan, phosphatidylserine, hydrogenated egg phosphatidylcholine, L-α egg phosphatidylcholine, dipalmitoylphosphatidylcholine, 1,2-diacyl-glycero-3-phosphocholine/poly(ethyleneglycol) 2000-1,2-diacyl-glycero-3-phosphoethanolamine/cholesterol, and distearoylphosphatidylcholine and hydrogenated phosphatidylinositol (18–20). Key characteristics of these liposomes include their decreased clearance and increased accumulation at affected organ sites. This system can therefore alter the biodistribution and pharmacokinetics of the encapsulated drug (21).

Once the drugs had been modified in the present study, the release efficiency was 65.1% after 48 h and the entrapping efficiency was 85%. Water-soluble drugs can be enfolded closely by the hydrophilic molecules of liposomes so that the drug molecules can be released slowly in the body. Overall, low drug loading and a low encapsulation efficacy are characteristics of conventional liposomes produced for cisplatin (22,23). Conventional liposomes are liposomes with varying lipid compositions. Compositions that are very high in phosphatidylcholine and cholesterol are typically the most commonly used (24). It has been suggested in previous studies that phosphatidylethanolamine transfers across mammalian cell membranes more easily than phosphatidylcholine (25). This may contribute to the strong ability to carry cisplatin into cells.

Liposomes targeting tumor cells have shown considerably greater cytotoxicity. This is due to an increased bioavailability once the liposomes have been transported to the cytoplasm, where the liposomal membrane is broken down by degradative enzymes and the drug is released (24). In the present study, the results showed that administering PL-CBP to SGC-7901 gastric tumor cell-bearing mice was therapeutically more effective and resulted in less side-effects compared with free CBP. Next, it was found that PL-CBP induced tumor cell apoptosis and resulted in accumulation of a large amount of Pt in the tumors. This may be the reason for the apparent antitumor effect in the PL-CBP group. Earlier studies demonstrated that the tumor accumulation of liposomal drugs correlated with their circulation time in the blood (25). Later studies found that liposome accumulation in tumors was not only correlated with blood circulation levels, but also with the size of the liposomes (26). Another study reported that doxorubicin encapsulation in 100-nm liposomes coated with PEG resulted in increased blood levels and an enhanced survival time in the circulation (25). The formulation used in the present study was by far the most efficient system, and increased the levels of CBP in the tumors. The main side-effects of CBP are its toxicity to the kidney and damage to the marrow, with severe thrombocytopenia and leucopenia (27). The results presented in the present study show that PL-CBP can increase bioavailability and may increase the therapeutic efficacy.

The extent of lymph node metastasis is the most important prognostic factor, as analyzing node metastasis has become the leading method for assessing the extent of disease, determining the prognosis in gastric cancer patients and affecting therapy strategies (28). In the present study, lymph node metastasis was evaluated according to the assessing standard (16). The results showed that the lymph node metastasis in the PL-CBP group was the lowest among the three groups.

Overall, the study showed that the characteristics of PL-CBP with PEG-2000 were stable and indicated an obvious antitumor and antimetastasis effect in the gastric cell-bearing mice. In future studies, we aim to further detect the biological mechanism behind the antitumor and antimetastasis effects of PL-CBP.

## Figures and Tables

**Table I:** Size and drug release of liposomal formulations^a^.

Time, h	Size, nm	Re, %
0	82.1±2.3	0.7±0.1
12	85.2±1.8	22.5±5.7
24	88.5±3.5	48.7±7.3
48	87.5±4.3	65.1±9.7

a Data are presented as the mean ± standard deviation of three independent measurements. Re release efficiency.

**Table II:** Volume and grade of lymph node metastasis^a^.

		Grade			
		
Group	Volume, mm^3^	0	+	++	+++
Control	98±54				10
PL-CBP	37±24^b^	6	4^b^		
CBP	12±10^b^		5	5^c^	

a Data are presented as the mean ± standard deviation of three independent measurements.

b P<0.001 and

c P<0.05 vs. control.

CBP, carboplatin; PL-CBP, polyethylene glycol (PEG)ylated CBP.

## Abbreviations

PEG: polyethylene glycol
PL-CBP: PEGylated carboplatin liposomes
CBP: carboplatin
PEG-2000: polyethylene glycol-2000
HE: hematoxylin and eosin
AAS: atomic absorption spectroscopy
TUNEL: terminal deoxynucleotidyl transferase-mediated dUTP nick end labeling
AI: apoptosis index
